# Neurological and growth outcomes in South African children with congenital cytomegalovirus: A cohort study

**DOI:** 10.1371/journal.pone.0238102

**Published:** 2020-09-17

**Authors:** Jayani Pathirana, Leanne Texeira, Hannah Munian, Firdose Nakwa, Ismail Mayet, Innocent Maposa, Michelle J. Groome, Suresh Boppana, Shabir A. Madhi

**Affiliations:** 1 Medical Research Council: Respiratory and Meningeal Pathogens Research Unit, Faculty of Health Sciences, University of the Witwatersrand, Johannesburg, South Africa; 2 Department of Science and Technology/ National Research Foundation: Vaccine Preventable Diseases, Faculty of Health Sciences, Johannesburg, University of the Witwatersrand, Johannesburg, South Africa; 3 Department of Speech Therapy and Audiology (STA), Chris Hani Baragwanath Academic Hospital (CHBAH), Diepkloof, Johannesburg, South Africa; 4 Department of Paediatrics, Chris Hani Baragwanath Academic Hospital, School of Clinical Medicine, Faculty of Health Sciences, University of the Witwatersrand, Johannesburg, South Africa; 5 Department of Ophthalmology, St. Johns Eye Hospital, Chris Hani Baragwanath Academic Hospital, School of Clinical Medicine, Faculty of Health Sciences, University of the Witwatersrand, Johannesburg, South Africa; 6 Department of Epidemiology and Biostatistics, School of Public Health, Faculty of Health Sciences, University of Witwatersrand, Johannesburg, South Africa; 7 Department of Pediatrics, University of Alabama School of Medicine, Birmingham, Alabama, United States of America; 8 Department of Microbiology, University of Alabama School of Medicine, Birmingham, Alabama, United States of America; University of St Andrews, UNITED KINGDOM

## Abstract

**Objectives:**

To assess neurological sequelae and growth in the first 12 months of life in a cohort of congenital cytomegalovirus (cCMV) infected infants compared to cCMV uninfected infants.

**Study design:**

This was a prospective matched cohort study conducted in Soweto, South Africa where forty-six confirmed cCMV cases were matched on HIV-exposure, gender and gestational age (±two weeks) to 84 cCMV-uninfected controls in a 1:2 ratio. Cases and controls were followed up until 12 months of age to assess anthropometry, hearing and neurodevelopmental outcomes.

**Results:**

Thirty-four (73.9%) cCMV cases and 74 (88.1%) controls, completed all assessments at 12 months age. At 12 months, one cCMV case had died, none of the children in either group had SNHL and neurodevelopmental delay was present in a similar percentage of cCMV cases (n = 2; 6%) and controls (n = 1, 4%; OR 1.09, 95% CI 0.04–27.84, p = 0.958). Anthropometry did not differ between cases and controls overall throughout the follow up period. HIV-exposed cases had smaller head circumference for age at 6 and 12 months when compared with HIV-exposed controls.

**Conclusion:**

By 12 months of age, there was no evidence of a difference in neurological sequelae between cCMV infected South African children and cCMV uninfected children in this study. Further follow-up is warranted to detect late-onset hearing loss and neurodevelopmental delay beyond 12 months of age.

## Introduction

Cytomegalovirus (CMV) is the most common congenital infection worldwide [1]. The majority of infants with congenital CMV (cCMV) are born to women with non-primary CMV infection [2, 3] in settings with high CMV seroprevalence, such as in most low-middle income settings [4].

Congenital CMV is symptomatic in 10–15% of infected infants and a significant proportion of these children develop CMV associated neurological sequelae including sensorineural hearing loss (SNHL, 30–60%), cerebral palsy and mental retardation (50–80%), and visual impairment (20–35%) [5, 6]. Most cCMV infections are clinically asymptomatic at birth (85–90%) [7], however, permanent neurological sequelae (13.5%) can develop during childhood [5] with SNHL (10%) being the most common [7]. Most studies reporting on outcomes of cCMV infection are from high-income, settings where cCMV is the result of maternal primary infection. Although most cCMV infection in sub-Saharan African populations are the result of maternal non-primary infection usually first acquired during childhood [8–10], population-based studies estimating neurological sequelae in cCMV infected children of mothers with non-primary CMV infection from African settings are limited.

Human immunodeficiency virus (HIV) infected infants with cCMV co-infection, in the pre-antiretroviral therapy (ART) era, had accelerated progression of HIV disease, neurological deterioration and increased mortality [11]. With the advent of prevention of mother to child HIV transmission (PMTCT) measures through lifelong ART for HIV-infected pregnant women, most children of HIV infected mothers are not themselves HIV infected. These HIV-exposed but uninfected children are at increased risk of mortality, growth and developmental deficits compared to HIV-unexposed children [12]. Whether these deficits are due to cCMV infection has not been characterized.

We previously reported a cCMV prevalence of 2.5% in South African children with higher prevalence in HIV-exposed (5.2%) than in in HIV-unexposed infants (1.4%) from low-middle income households [13]. The aim of this study was to compare hearing, neurodevelopmental and growth outcomes in this cohort of cCMV-infected children with a group of cCMV-uninfected matched controls until 12 months of age.

## Patients and methods

### Study design and population

This was a prospective matched cohort study, and the study population and participant screening and enrolment have been previously described [13]. In brief, the study was conducted in Soweto, which is the largest urban settlement in South Africa, constituting almost exclusively of Black Africans of multiple tribal ethnicities. It is a low-middle income setting with the majority of births (95.0%) occurring at public health facilities and three quarters of these births taking place at Chris Hani Baragwanath Academic Hospital (CHBAH), a secondary and tertiary care hospital. Neonates born at CHBAH (N = 2685) were screened for cCMV from May to December 2016 by testing saliva collected within 72 hours of birth using a real-time Polymerase Chain Reaction (PCR) assay as described [14]. Neonates with positive saliva specimens for CMV at birth, had additional saliva and/or urine tested for CMV within 21 days of birth for infection confirmation. Infants with confirmed cCMV (referred to as cases) were matched to a group of healthy CMV uninfected infants (referred to as controls) who screened negative for CMV in saliva at birth and on repeat saliva testing within 21 days of birth without a history of hospitalization after birth. Cases were matched to controls on gender, gestational age (±two weeks) and exposure to maternal HIV *in-utero* and enrolled in a prospective study to determine hearing, neurodevelopmental and growth outcomes during the first 12 months of life (ClinicalTrials.gov Identifier: NCT03722615). Cases were additionally assessed for symptomatic cCMV infection between birth and 21 days of birth.

**Fig 1:**
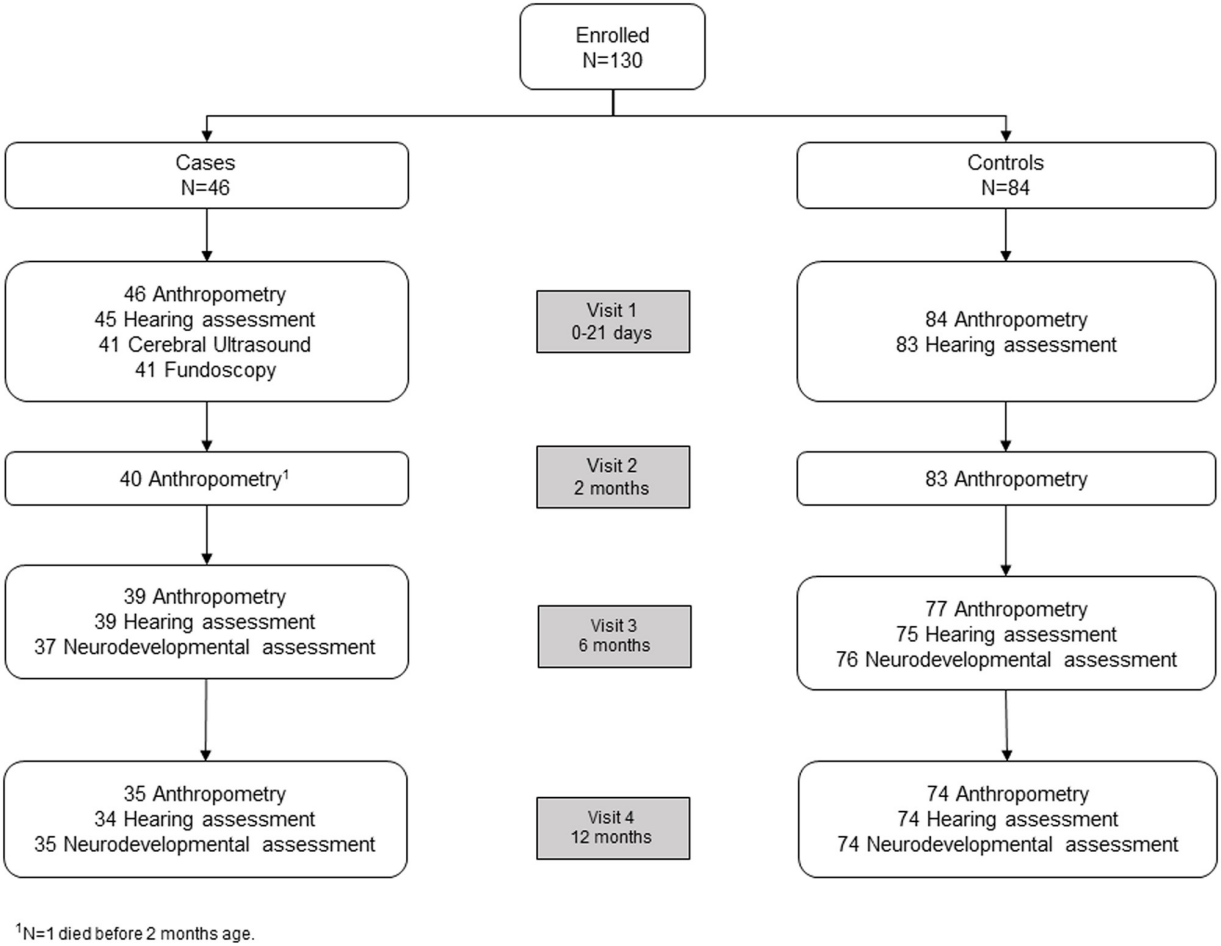
Flow chart representing numbers of cases and controls enrolled and assessments at each study visit.

The HIV status of infants were classified as HIV-unexposed (HIV uninfected infants born to HIV uninfected mothers), HIV-exposed (HIV uninfected infants born to HIV infected mothers) or HIV infected (HIV infected infants born to HIV infected mothers). Participant enrolment and assessments are shown in Fig 1.

As described previously, maternal CMV seroprevalence was determined in mothers of confirmed cases and controls by testing serum collected at delivery for anti-CMV Immunoglobulin G (IgG) and Immunoglobulin M (IgM) to determine the proportion of infants born to women with non-primary CMV infection [13].

### Anthropometry

Birth weight was extracted from neonatal records. Head circumference, mid-upper arm circumference (MUAC), length (Seca 416 infantometer) and weight (SECA 354 digital baby scale from zero to six months of age and Seca 876 2-in-1 scale from six to 12 months of age) were measured at each of four study visits which included, within 21 days of birth and at two, six and 12 months of age by the study physician or nurse (Fig 1). Anthropometry was converted to standardized Z-scores using the WHO Child Growth Standards [15]. Conversion of anthropometry to Z-scores was not possible for preterm infants until corrected age surpassed term gestation.

### Clinical data

Maternal and infant medical records were reviewed for clinical data, HIV PCR results, ART duration, birth weight and any hospital admission and investigation. Symptomatic cCMV infection at birth was defined as one or more findings of jaundice with conjugated hyperbilirubinemia>2mg/dL, thrombocytopenia (platelet count <100 000/mm^3^), petechiae, microcephaly, hepatosplenomegaly, seizures and/or chorioretinitis [16].

### Ophthalmologic examination

Only cCMV infected cases had an examination of the retina by direct fundoscopy within 21 days of birth by the ophthalmologist at CHBAH as part of the assessment for symptomatic cCMV. Ophthalmological features of CMV disease was defined as fundus abnormalities include chorioretinitis with or without hemorrhage, peripheral retinal scars, optic atrophy, and macular scars, anterior segment abnormalities or strabismus [17]. Follow-up care was provided as appropriate if abnormalities were detected. We did not perform ophthalmologic examination in controls as they were assessed to be healthy and CMV uninfected at birth and to avoid unnecessary investigations in healthy children in a resource limited setting.

### Cerebral ultrasonography

Cases of cCMV had cerebral ultrasonography performed by neonatologists within 21 days of birth at a cerebral sonar clinic at CHBAH to describe any lesions that may be associated with cCMV as an exploratory objective. Cerebral findings was not included in the definition of symptomatic cCMV. Infants with ultrasound abnormalities were referred to a specialist neonatology clinic for care and management. Cerebral ultrasonography was defined as abnormal if intracranial calcification, pseudocysts or cerebellar hypoplasia were identified [18].

### Hearing assessment

Audiologists at CHBAH conducted the hearing assessments on all cases and controls. Within 21 days of birth, infants received hearing screening by automated auditory brainstem response (AABR [Maico MB11 BERAphone^®^, MAICO Diagnostics GmbH]), with integrated electrodes at an intensity of 35dBnHL. At age six and 12 months, screening included ipsilateral acoustic reflex thresholds, distortion product otoacoustic emissions (DPOAE) and AABR screening. A hearing screening ‘pass’ during the neonatal period included pass AABR bilaterally. A hearing screening ‘pass’ at six and 12 months included either: 1) pass AABR bilaterally, or 2) acoustic reflexes present in at least two frequencies in both ears and three screening DPOAEs present at three frequencies in both ears. Diagnostic hearing assessments for children not passing hearing screening (classified as “referred” hearing screening) included otoscopy, tympanometry, electrophysiological measures (i.e. diagnostic auditory brainstem responses, auditory steady state responses, cochlear microphonic testing), and diagnostic otoacoustic emissions (OAE) and behavioral audiometric evaluations as developmentally appropriate. Audiological management and referral to the pediatric ear nose and throat specialists took place when indicated. SNHL was defined as unilateral SNHL with thresholds greater than or equal to 25 decibels hearing level (dBHL) or bilateral SNHL with thresholds greater than or equal to 25 dBHL in the better ear. Severity of hearing loss was categorized as: mild (26 to 40 dBHL), moderate (>41–55dBHL), moderately severe (56-70dBHL), severe (71–90dBHL), or profound (>90dBHL) [19].

### Neurodevelopmental assessment

The Bayley III scales of infant and toddler development was administered to all cases and controls at six and 12 months of age by the study physician. Each neurodevelopmental domain was assessed based on observed responses to a set of tasks presented to the child and scored directly on the following subscales: cognitive scale, language summed scale of receptive and expressive language subscales and motor summed scale of fine and gross-motor subscales. Composite scores were derived from raw scores for cognitive, language, and motor development and scaled to a metric, with a mean of 100, standard deviation of 15, and range of 40 to 160. Performance that was 2.0 or more standard deviations below the mean score in any domain was defined as neurodevelopmental delay [20, 21].

#### Neurological sequelae

Neurological sequelae was defined as a composite of SNHL and neurodevelopmental delay at 12 months of age.

### Statistical analysis

The sample size of cases and controls required to detect any difference in neurological sequelae was based on the expected prevalence of neurological sequelae in cCMV infected and uninfected children from the available literature. The prevalence of neurological sequelae in cCMV infected infants was expected to be approximately 17% [5]. The anticipated prevalence of neurological sequelae in cCMV uninfected controls with uneventful fetal or perinatal periods was estimated at 1% [22]. For 80% power, allowing for a loss to follow-up of 10%, 42 cCMV infected cases and 84 uninfected controls (n = 126) with an allocation ratio of 1:2 were enrolled.

Data were analyzed using Stata 13.0 (Stata Corp, College Station, TX) and p-value of <0.05 was considered statistically significant.

Symptomatic cCMV infection in cases was described based on clinical findings at birth and ophthalmologic examination. The difference in symptoms between HIV-exposed and HIV-unexposed cases were compared by Fisher’s exact test. Cerebral ultrasound findings in cases were described and a comparison made between HIV-exposed and HIV-unexposed cases using the Fisher’s exact test.

Hearing results during the neonatal period, and at six and 12 months of age and neurodevelopmental assessment results using the Bayley III scoring at six and 12 months of age were compared between cases and controls by conditional logistic regression and odds ratios computed and further analyzed stratified by HIV-exposure. Neurodevelopmental analysis was additionally adjusted for comorbidities (one case with congenital heart disease).

A multivariable linear mixed effects model was used to estimate the differences in anthropometric Z-scores (based on the World Health Organization [WHO] 2006/2007 growth standards) between cases and controls in general and marginal effects at each study visit in particular with the model adjusted for breastfeeding and HIV infection. In addition, as the analysis considered growth over time, microcephaly present at birth was adjusted for. Individual infant variability in the measurements that were done at different follow-up times were accounted for using random intercept and random slope. For the group matching variable, we assumed only a random intercept. The mean differences between anthropometric Z-scores between HIV-exposed cases and controls and HIV-unexposed cases and controls were also analyzed. All tests were 2-sided with a significance level of 5%.

### Ethical approval and participant consent

The Human Research Ethics Committee of the University of Witwatersrand approved this study (Proposal number: M151161). Mothers provided written informed consent for screening neonates for cCMV, follow-up and assessments. The study was registered on clinicaltrials.gov (NCT03722615).

## Results

From May to December 2016, 130 infants were enrolled at birth including 46 cCMV cases and 84 controls (Fig 1 and Table 1). All infants were Black South Africans except for one control infant of mixed race, whose mother was mixed race and father was Asian. Of HIV-exposed infants (N = 77), five were in utero HIV-infected and initiated on appropriate anti-retroviral therapy. CD4 counts and HIV viral load were not available for these infants. These five infants were also cCMV-infected (p = 0.002), Table 1. Most of the cases and controls (94.6%) were born to women with non-primary CMV infection (IgG positive). The type of maternal CMV infection was undetermined in seven (5.3%, 7/130) women who were both IgG and IgM positive with 42.8% (3/41) mothers to cCMV cases and 57.1% (4/7) mothers to cCMV uninfected controls (p = 0.561).

**Table 1:** Baseline characteristics cCMV infected cases and CMV uninfected controls.

	Cases	Controls	p-value
	N = 46	N = 84	
**Female**^1^**, n (%)**	21 (45.7)	41 (48.8)	0.730
**Birth weight (g), median (IQR)**	2845 (2455–3190)	2902.5 (2595–3195)	0.411
**Gestational age**^1^ **(weeks), median (IQR)**	38 (36–40)	38 (36–39)	0.897
**Small for gestational age, n (%)**	10 (22)	9 (11)	0.100
**HIV-exposed**^1^**, n (%)**	28 (60.9)	49 (58.3)	0.641
HIV-infected, n (%)	5 (11.1)	0 (0)	0.002
Duration of Maternal ARV prior to delivery, mean days (SD)	316.5 (461.6)	530.7 (815.9)	0.260
**Maternal age at delivery (years), median (IQR)**	25 (22–29)	28 (24–33)	0.021
**Breastfeeding, n (%)**	35 (76.1)	62 (73.8)	0.775
**Mortality, n (%)**	1 (2.2%)	1 (1.2)	0.327

^1^ Cases and controls were matched on gender, gestational age and HIV-exposure and there are no differences expected in these variables between cases and controls.

At the age of 12 months, 34 (74%) cases and 74 (88%) controls completed all assessments with an attrition rate of 17% and 12% (p = 0.684), respectively, resulting in 74% power to detect 17% difference in neurological sequelae between the groups (assuming a rate of 1% in controls). There was no difference in baseline characteristics at birth between infants that dropped out and those remaining in the study (data not shown).

### Symptomatic cCMV infection

Three (7%) cCMV cases were symptomatic at birth. One each had microcephaly (HIV-infected), neonatal jaundice with conjugated hyperbilirubinemia>2mg/dL, (HIV-unexposed) and thrombocytopenia with platelet count <100 000/mm^3^ (HIV-unexposed). One (2%) asymptomatic cCMV case, co-infected with HIV *in-utero*, died at the age of two months due to meningitis of unknown etiology. All had normal retinal examinations except for one with retinopathy of prematurity, born at 28 weeks gestation, which resolved by six months of age. One case had cardiac surgery for Tetralogy of Fallot at the age of six months. There was no difference in symptoms between HIV-exposed and HIV-unexposed cases.

### Cerebral ultrasound

Forty-one cCMV cases had cerebral ultrasound scans of whom six (15%) had abnormalities detected. Abnormalities included three (7%) with cysts, two (5%) with ventricular dilatation and one (2%) with calcifications. There was no significant difference in cerebral abnormalities between HIV-exposed (21%) and HIV-unexposed cases (6%; p = 0.373). The case with microcephaly, could not be assessed successfully due to fusion of the fontanelles. Three cases were initiated on oral valganciclovir (one symptomatic case with microcephaly and two asymptomatic cases with abnormal cerebral imaging) based on the attending neonatologist’s clinical judgement.

### Audiologic outcome

Among the 130 infants enrolled, 45 cases and 83 controls had hearing screening in the neonatal period (Table 2). Thirty-nine cases (86.7%) and 76 controls (91.6%) passed hearing screening suggesting intact auditory neural pathways at birth with no significant difference between cases and controls (Odds Ratio [OR] 1.6; 95% confidence interval [95% CI] 0.5–5.3; p = 0.385). Of infants that did not pass screening, six (13.3%) were cases and seven (8.4%) were controls. Of these infants that completed repeat testing at two months of age, all had evidence of middle ear dysfunction and SNHL could not be completely assessed.

**Table 2:** Hearing screening results of congenital CMV infected and uninfected children over 12 months.

	Total	CMV infected cases	CMV uninfected controls	Odds Ratio^1^ (95% CI)	P-value
		N = 46	N = 84		
**Newborn screening**^2^	n = 128	n = 45	n = 83		
Passed, n (%)	115 (89.8)	39 (86.7)	76 (91.6)	1.6 (0.5–5.3)	0.385
Referred, n (%)	13 (10.2)	6 (13.3)^3^	7 (8.4)^4^		
**6 months screening**^5^	n = 113	N = 38	n = 75		
Passed, n (%)	78 (69.0)	31 (81.6)	48 (64.0)	0.5 (0.2–1.3)	0.170
Referred, n (%)	36 (31.9)	7 (18.4)^6^	27 (36.0)^7^		
**12 months screening**^5^	n = 108	n = 34	n = 74		
Passed, n (%)	89 (82.4)	31 (91.2)	58 (78.3)	0.5 (0.1–1.8)	0.292
Referred, n (%)	19 (17.6)	3 (8.8)^8^	16 (21.6)^9^		

^1^Conditional logistic regression.

^2^AABR—Automated Auditory Brainstem Response.

^3^Two cases dropped out before further testing, 4 cases had evidence of middle ear dysfunction on repeat testing at two months of age.

^4^One control dropped out before further testing, six controls had evidence of middle ear dysfunction on repeat testing at two months of age.

^5^ART (acoustic reflex threshold), DPOAE (distortion product optoacoustic emissions), Otoscopy, Immittance.

^6^Five cases had evidence of middle ear effusion or dysfunction. Two children did not complete testing despite repeated attempts.

^7^26 had evidence of middle ear effusions or dysfunction, one child could not complete testing despite repeated attempts.

^8^Three cases had evidence of middle ear dysfunction.

^9^12 had evidence of middle ear dysfunction, three (19%) did not cooperate with testing on two separate attempts and one had suspected hearing loss of uncertain etiology who did not return for additional testing.

At six months of age, 38 cases and 75 controls completed hearing assessments of whom 31 (81.6%) cases and 48 (64.0%) controls had normal hearing with no significant difference between cases and controls (OR 0.5; 95% CI 0.2–1.3; p = 0.170), Table 2. Of the seven (18.4%) cases and 27 controls (36.0%) that did not pass, five (71.4%) cases and 26 (96.3%) controls had evidence of middle ear effusion or dysfunction, detected during the hearing assessment.

At 12 months of age, 34 cases and 74 controls completed hearing assessments of whom 31 (91%) cases and 58 (78%) controls had normal assessments with no significant difference between cases and controls (OR: 0.5; 95% CI: 0.1–1.8; p = 0.292); Table 2. All three (9%) cases with abnormal assessment, had evidence of middle ear dysfunction. Of the 16 (22%) controls that did not pass, 12 (75%) had evidence of middle ear dysfunction, three (19%) did not cooperate with testing on two separate attempts and one had suspected hearing loss of uncertain etiology who did not return for additional testing.

There was no difference in hearing outcomes between HIV-exposed and HIV-unexposed cases at both six and 12 months of age.

### Neurodevelopmental outcome

At age six months, one (2.7%) symptomatic cCMV case, born with microcephaly, and none of the controls had neurodevelopmental delay. At 12 months of age two (5.7%) cases, one with symptomatic cCMV (microcephaly) and one with asymptomatic cCMV, and three (4%) controls had neurodevelopmental delay (OR 1.09, 95% CI 0.04–27.84, p = 0.958). The symptomatic case with microcephaly had delay across all three domains while the asymptomatic case, only had motor delay. Of the three controls, two had language delay and one had motor delay. A comparison of Bayley III neurodevelopmental assessment scores between cases and controls at six and 12 months is provided in S1 Table. Four (80.0%) of the five children with neurodevelopmental delay were HIV-exposed, although this was not significant (p = 0.192). There was no significant difference in neurodevelopmental outcome between HIV-exposed cases and controls and HIV-unexposed cases and controls at both six and 12 months of age.

A composite of neurodevelopmental delay and SNHL as neurological sequelae were analyzed between cases and controls at the age of 12 months. There was no difference in overall neurological sequelae between cases (2/35, 6%) and controls (3/74, 4%, OR 4.0, 95% CI 0.36–44.11, p = 0.258) at 12 months of age. There was no difference in clinical outcomes between valganciclovir treated and untreated cCMV cases.

### Anthropometry

**Fig 2:**
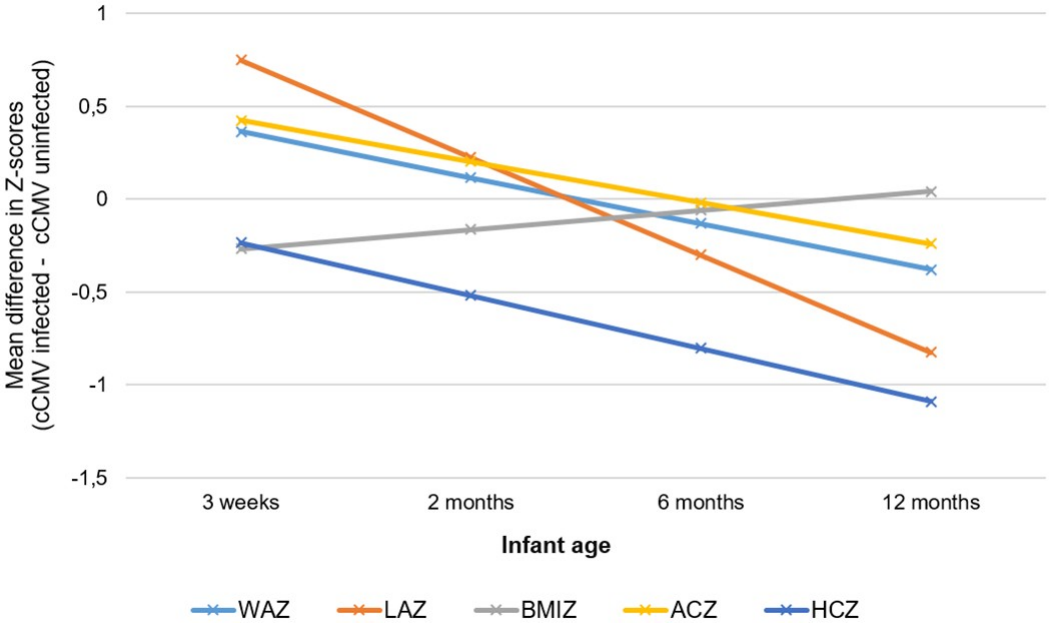
Trend in difference of mean anthropometry Z-scores between HIV-exposed congenital cytomegalovirus infected and uninfected children over 12 months. cCMV (congenital cytomegalovirus), WAZ (weight for age), LAZ (length for age), BMIZ (Body mass index for age), ACZ (arm circumference for age), Head circumference for age (HCZ).

**Fig 3:**
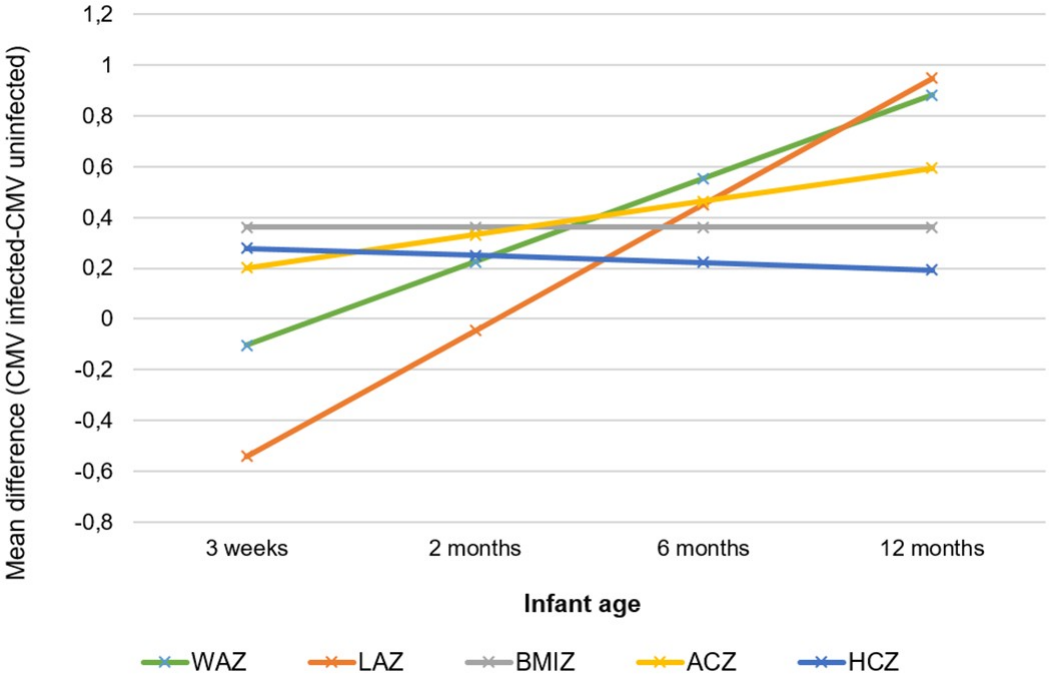
Trend in difference of mean anthropometry Z-scores between HIV-unexposed congenital cytomegalovirus infected and uninfected children over 12 months. cCMV (congenital cytomegalovirus), WAZ (weight for age), LAZ (length for age), BMIZ (Body mass index for age), ACZ (arm circumference for age), Head circumference for age (HCZ).

The mean raw anthropometry and standardized Z-scores are shown in S1 Table. Over the follow-up period, there was no significance in the mean difference in standardized scores for weight, length, MUAC, head circumference and BMI between cases and controls after adjusting for breastfeeding, HIV infection and microcephaly at birth. When stratified by HIV-exposure, the mean difference in standardized score for head-circumference was significantly lower at six (p = 0.02) and 12 months (p = 0.018) age in HIV-exposed cases than HIV-exposed controls (Table 3 and Fig 2). Table 3 shows the mean of the difference in Z-scores for anthropometry at each visit between HIV-exposed cases and controls and HIV-unexposed cases and controls with confidence intervals. Fig 2 shows the trend in the mean difference for each anthropometric Z-score over the four study visits. The mean difference in standardized scores for weight and length at 12 months of age were higher in HIV-unexposed cases than HIV-unexposed controls at 12 months (p = 0.013 and 0.043 respectively); Table 3 and Fig 3.

**Table 3:** Difference in mean anthropometry Z-scores between cCMV infected cases and cCMV uninfected controls over 12 months by HIV-exposure status.

	Age	WAZ^1^	p-value	LAZ^1^	p-value	BMIZ^1^	p-value	ACZ^1^	p-value	HCZ^1^^,^^2^	p-value
		Mean (95% CI)		Mean (95% CI)		Mean (95% CI)		Mean (95% CI)		Mean (95% CI)	
**Difference in mean anthropometry between HIV exposed cases and HIV-exposed controls**^3^	**3 weeks**	0.4 (-0.35, 1.08),	0.316	0.748 (-0.03, 1.53)	0.060	-0.267 (-1.08, 0.55)	0.519	0.424 (-0.84, 1.68)	0.509	-0.222 (-0.84, 0.39)	0.481
	**2 months**	0.1 (-0.49, 0.72),	0.706	0.223 (-0.35, 0.80)	0.447	-0.164 (-0.82, 0.49)	0.622	0.203 (-0.65, 1.06)	0.642	-0.464 (-0.99, 0.06)	0.081
	**6 months**	-0.132 (-0.76, 0.50)	0.683	-0.301 (-0.92, 0.31)	, 0.338	-0.061 (-0.71, 0.59)	0.853	-0.019 (-0.56, 0.52)	0.945	-0.706 (-1.30, -0.11)	0.020
	**12 month**	-0.380 (-1.17, 0.41)	0.344	-0.825 (-1.69, 0.04)	, 0.061	0.042 (-0.76, 0.84)	0.918	-0.241 (-0.77, 0.29)	0.371	-0.948 (-1.73, -0.16)	0.018
**Difference in mean anthropometry between HIV unexposed cases and HIV-unexposed controls**^4^	**3 weeks**	-0.104 (-0.74, 0.53)	0.749	-0.541 (-1.54, 0.46)	0.289	0.362 (-0.60, 1.32)	0.460	0.201 (-0.99, 1.39)	0.740	0.278 (-0.27, 0.83)	0.317
	**2 months**	0.225 (-0.32, 0.77)	0.415	-0.045 (-0.73, 0.64)	0.898	0.361 (-0.27, 0.99)	0.262	0.332 (-0.50, 1.16)	0.434	0.250 (-0.26, 0.76)	0.337
	**6 months**	0.554 (-0.01, 1.12)	0.055	0.452 (-0.19, 1.10)	0.170	0.361 (-0.27, 0.99)	0.264	0.463 (-0.10, 1.02)	0.106	0.222 (-0.33, 0.77)	0.427
	**12 months**	0.883 (0.19, 1.58)	0.013	0.949 (0.03, 1.87)	0.043	0.361 (-0.27, 0.99)	0.462	0.594 (0.06, 1.13)	0.029	0.194 (-0.45, 0.84)	0.555

Multivariable linear mixed effects model adjusting for breastfeeding and HIV infection.

^1^WAZ (weight for age); LAZ (length for age); BMIZ (Body mass index for age); ACZ (arm circumference for age); Head circumference for age (HCZ).

^2^Head circumference analysis adjusted for microcephaly at birth.

^3^Number of HIV-exposed cases and HIV-exposed controls compared at each age: 3 weeks of age: 28 cases and 49 controls; 2 months of age: 26 cases and 49 controls, 6 months of age: 24 cases and 45 controls; 12 months of age: 27 cases and 47 controls.

^4^ Number of HIV-unexposed cases and HIV-exposed controls at each age: 3 weeks of age: 18 cases and 35 controls; 2 months of age: 17 cases and 34 controls, 6 months of age: 17 cases and 34 controls; 12 months of age: 17 cases and 33 controls.

## Discussion

In this study, clinically symptomatic infection was present in 7% of cCMV infected newborns, which is lower than reported in other studies of 10–15% [1, 5, 7]. On follow-up, there was no difference in neurological sequelae and anthropometry between cCMV cases and controls by 12 months of age.

The definition of symptomatic infection varies among different studies. We did not consider small-for-gestational age (SGA) and presence of cerebral ultrasound abnormalities as symptomatic infection due to a lack of data on maternal conditions that may have resulted in SGA infants and because we did not investigate other causes for cerebral abnormalities, intended as a descriptive analysis. The symptomatic (microcephaly) cCMV case was co-infected with HIV and the resulting immunosuppression may have contributed to severe CMV disease during the foetal period with brain involvement. The fewer number of symptomatic cases in our study could be likely due to all cCMV infections following non-primary maternal infection, though recent evidence has shown similar rates of cCMV disease between infants born to women with primary and non-primary CMV infection [23]. Underlying differences in population characteristics and other yet to be identified factors may play a role in the frequency and severity of symptomatic cCMV at birth.

None of the cCMV-infected infants who completed assessments had SNHL. Late onset SNHL may manifest after 12 months of age as described by others [2, 24, 25]. A Brazilian study, also from a high CMV sero-immune population, reported SNHL prevalence of 8.6% (5/58, 95% CI 2.9–19.0) in cCMV infected children at a median age of 24 months (range 15–50 months) [2]. In a Japanese study, one (2.3%) of 43 asymptomatic cCMV cases at birth developed SNHL by 12 months of age compared to 12 (70.6%) of 17 symptomatic children [24]. Three children in our study also received oral valganciclovir therapy, which has been shown to improve hearing outcomes [26]. The high prevalence of middle ear disease interfered with testing to reliably document SNHL in both cases and controls.

There was no significant difference in neurodevelopment between cases and controls at 12 months of age. Due to the limited number of symptomatic cCMV, it was not possible to reliably compare difference in neurodevelopmental outcome between symptomatic and asymptomatic cCMV infection. Prospective studies of asymptomatic cCMV infected children with follow-up of one to six years report a cumulative incidence of neurodevelopmental impairment between 0% to 9.1% [27]. In contrast, studies of symptomatic newborns report a prevalence of 30–50% neurological impairment during childhood [1, 5, 28]. Some studies have found neurological sequelae to be present only in symptomatic children with obvious brain involvement [29], however these were mainly from the Americas and Europe where the epidemiology of cCMV likely differs from our population.

Standardized anthropometry were not significantly different between cCMV cases and controls over the 12 months of follow-up. When stratified by HIV-exposure, cCMV infected, HIV-exposed children had lower standardized head-circumference for-age than HIV-exposed children without cCMV. HIV-unexposed cCMV infected children however, had higher standardized weight-for-age and length-for-age scores than their controls. Although our numbers for the stratified analysis are low, similar findings have been observed in Zambian HIV-exposed and HIV-unexposed, CMV infected (congenital or acquired) children [9]. Several mechanisms in which CMV affect growth and development have been proposed which may be both disruptive and beneficial for growth. CMV infects all cell types with the resulting inflammation possibly disrupting cell growth [18], and may affect absorption of nutrients by affecting gut microbiota [9, 30], which may be more marked in HIV-exposed children. Conversely, latent herpesvirus infections such as CMV have beneficial immune modulation and outcome in mouse models with resistance to some bacterial pathogens [31]. Whether this occurs in human infants with resulting advantages for growth, requires further study. The observation in our study of more frequent middle ear infections in cCMV uninfected controls than cases is of interest in this regard.

Limitations in our study included the higher than anticipated drop-out rate in both cases and controls which may have resulted in missing some cases with neurological sequelae and the lack of a significant difference in neurological sequelae between cases and controls. As there was no difference in baseline characteristics between drop outs and infants remaining in the study there is however unlikely to be selection bias. The cerebral ultrasound scans were conducted by different neonatologists, as an exploratory investigation, which may have led to inter-observer bias in identifying abnormalities. We followed up infants until 12 months of age, whereas longer follow up may have enabled identification of late onset sequelae.

## Conclusion

In this study, there was no evidence of a difference in neurological sequelae between cCMV infected children and a control group of cCMV uninfected children by 12 months of age in a study population from Soweto. Given the high prevalence of cCMV in the black South African population and HIV-exposed children, long-term follow-up throughout childhood is necessary to determine the burden of late-onset neurological sequelae and the need for a universal or targeted birth CMV screening program.

## Supporting information

S1 Table Comparison of Bayley III neurodevelopmental assessment scores between cases and controls at six and twelve months. (DOCX)

S2 Table Mean and standardized anthropometry of cases and controls at each visit. (DOCX)

## Abbreviations

AABR: Automated auditory brainstem response
ART: Antiretroviral therapy
cCMV: Congenital cytomegalovirus
CHBAH: Chris Hani Baragwanath Academic Hospital
CMV: Cytomegalovirus
dBHL: Decibels hearing level
DPOAE: Distortion product otoacoustic emissions
HIV: Human immunodeficiency virus
MUAC: Mid-upper arm circumference
OAE: Otoacoustic emissions
OR: Odds ratio
SARChI: The South African Research Chairs Initiative
SNHL: Sensorineural hearing loss
WHO: World Health Organisation
