# Supplementary material for: Neurological and growth outcomes in South African children with congenital cytomegalovirus: A cohort study
Source: PLoS One. 2020 Sep 17;15(9):e0238102. doi: 10.1371/journal.pone.0238102 (PMC7498063; doi:10.1371/journal.pone.0238102)
Supplement: S1 Table — (DOCX) [file pone.0238102.s001.docx]

###### **S1 Table: Comparison of Bayley III neurodevelopmental assessment scores between cases and controls at six and twelve months**

| **Bayley III Domain** | **6 months of age** | | | | **12 Months of age** | | | |
| --- | --- | --- | --- | --- | --- | --- | --- | --- |
|  | **cCMV**  **cases** | **Controls** | **Odds ratio^1^**  **(95% CI)** | **P- value** | **cCMV cases** | **Controls** | **Odds ratio^1^**  **(95% CI)** | **P- value** |
| **Cognition composite** | N=37 | N=76 |  |  | N=35 | N=74 |  |  |
| Mean (SD) | 102.5 (11.3) | 105.1 (8.4) | 0.98  (0.93, 1.03) | 0.459 | 107 (12.0) | 106.1 (8.5) | 1.04  (0.99, 1.11) | 0.137 |
| Range | 55-120 | 80-115 |  |  | 55-125 | 80-120 |  |  |
| **Language composite** | N=37 | N=76 |  |  | N=35 | N=74 |  |  |
| Mean (SD) | 93.7 (8.7) | 96.2 (6.6) | 0.97  (0.91, 1.03) | 0.327 | 94.3 (13.5) | 94.8 (10.0) | 1.0  (0.95, 1.04) | 0.924 |
| Range | 56-112 | 77-112 |  |  | 47-118 | 65-112 |  |  |
| **Receptive scaled** | N=37 | N=76 |  |  | N=35 | N=74 |  |  |
| mean (SD) | 9.1 (1.3) | 9.5 (1.5) | 0.85  (0.61, 1.19) | 0.345 | 9.6 (2.6) | 9.6 (2.2) | 1.0  (0.80, 1.23) | 0.947 |
| Range | 5-11 | 7-12 |  |  | 1-13 | 4-14 |  |  |
| **Expressive scaled** | N=37 | N=76 |  |  | N=35 | N=74 |  |  |
| mean (SD) | 8.8 (2.0) | 9.2 (1.3) | 0.90  (0.68, 1.19) | 0.461 | 8.4 (2.3) | 8.6 (1.57) | 0.99  (0.74, 1.32) | 0.925 |
| Range | 0-13 | 5-13 |  |  | 1-13 | 2-11 |  |  |
| **Motor composite** | N=37 | N=76 |  |  | N=35 | N=74 |  |  |
| Mean (SD) | 104.2 (13.9) | 106.1 (12.0) | 0.99  (0.96, 1.03) | 0.756 | 103.1 (14.7) | 105.7 (12.1) | 0.99  (0.95, 1.04) | 0.783 |
| Range | 46-121 | 76-136 |  |  | 46-130 | 67-130 |  |  |
| **Fine motor scaled** | N=37 | N=76 |  |  | N=35 | N=74 |  |  |
| Mean (SD) | 11.3 (2.6) | 11.6 (1.9) | 0.98  (0.79, 1.20) | 0.825 | 11.2 (2.5) | 11.7 (2.3) | 0.94  (0.73, 1.20) | 0.608 |
| Range | 1-15 | 8-16 |  |  | 1-15 | 6-19 |  |  |
| **Gross motor scaled** | N=37 | N=76 |  |  | N=35 | N=74 |  |  |
| Mean (SD) | 10.0 (2.5) | 10.4 (2.8) | 0.95  (0.80, 1.14) | 0.604 | 9.7 (2.8) | 10.1 (2.9) | 0.99  (0.83, 1.19) | 0.926 |
| Range | 1-13 | 3-19 |  |  | 1-15 | 1-16 |  |  |

^1^ Odds ratios derived from conditional logistic regression adjusted for comorbidities
