# Supplementary material for: Neurological and growth outcomes in South African children with congenital cytomegalovirus: A cohort study
Source: PLoS One. 2020 Sep 17;15(9):e0238102. doi: 10.1371/journal.pone.0238102 (PMC7498063; doi:10.1371/journal.pone.0238102)
Supplement: S2 Table — (DOCX) [file pone.0238102.s002.docx]

## **S2 Table: Mean and standardized anthropometry of cases and controls at each visit**

|  | **Weight (kg)** | | **Length (cm)** | | **Arm circumference (cm)** | | **Head circumference (cm)** | | **BMI** | |
| --- | --- | --- | --- | --- | --- | --- | --- | --- | --- | --- |
|  | **CMV +** | **CMV-** | **CMV+** | **CMV-** | **CMV+** | **CMV-** | **CMV+^1^** | **CMV-** | **CMV+** | **CMV-** |
| **Visit 1** | N=46 | N=84 | N=46 | N=84 | N=46 | N=83 | N=46 | N=84 | N=46 | N=84 |
| Mean (SD) | 2.96 (0.513) | 3.28 (0.612) | 49.6 (4.2) | 50.9 (3.6) | 9.9 (0.8) | 10.2 (1.3) | 34.7 (1.8) | 35.5 (1.8) | 14.4 (3.1) | 14.7 (2.7) |
| Range | 1140-3770 | 2.02-4.81 | 41-61 | 36.5-58 | 8.4-11.5 | 6.8-13.4 | 28.3-37.7 | 29.8-38.2 | 5.9-19.3 | 8.3-20.0 |
| **Visit 1^2^** | N=37 | N=78 | N=37 | N=78 | NA | NA | N=37 | N=78 | N=37 | N=78 |
| Z-score Mean (SD) | -1.0 (1.0) | -0.9 (1.0) | -0.5 (2.2) | -0.4 (1.5) | NA | NA | -0.13 (1.2) | 0.1 (1.2) | -1.0 (1.9) | -0.9 (1.3) |
| **Visit 2** | N=40 | N=83 | N=40 | N=83 | N=40 | N=82 | N=40 | N=82 | N=40 | N=83 |
| Mean (SD) | 5.22 (0.894) | 5.13 (0.994) | 58.2 (3.6) | 56.9 (3.7) | 12.4 (1.1) | 12.3 (1.0) | 39.4 (2.2) | 39.7 (1.8) | 15.4 (2.5) | 15.3 (3.8) |
| Range | 3.51-7.60 | 1.08-7.13 | 51.4-65.3 | 40.8-68 | 10.3-15 | 9.1-15 | 32.2-46 | 34.8-45 | 10.8-21.5 | 4.9-37.7 |
| Z-score Mean (SD) | -0.6 (1.1) | -0.7 (1.4) | -0.2 (1.5) | -0.8 (1.7) | NA | NA | 0.3 (1.8) | 0.6 (1.2) | -0.6 (1.2) | -0.3 (2.2) |
| **Visit 3** | N=39 | N=77 | N=39 | N=77 | N=39 | N=77 | N=39 | N=77 | N=39 | N=77 |
| Mean (SD) | 7.46 (1.306) | 7.31 (1.068) | 65.9 (2.9) | 66.2 (2.6) | 13.7 (1.3) | 13.6 (1.0) | 43.0 (2.1) | 43.9 (1.7) | 16.3 (2.9) | 15.6 (2.4) |
| Range | 5.4-10.9 | 5.4-9.7 | 59.3-72.2 | 59-73 | 11.4-17.4 | 11.6-16.5 | 35.6-48 | 37.8-48.7 | 9.9-22.8 | 9.2-22.0 |
| Z-score Mean (SD) | -0.3 (1.5) | -0.5 (1.1) | -0.4 (1.3) | -0.4 (1.1) | -0.3 (1.2) | -0.4 (0.9) | 0.1 (1.6) | 0.8 (1.3 | -0.1 (1.6) | -0.4 (1.2) |
| **Visit 4** | N=35 | N=74 | N=35 | N=74 | N=35 | N=74 | N=35 | N=74 | N=35 | N=74 |
| Mean (SD) | 9.2 (1.6) | 9.1 (1.3) | 72.9 (3.3) | 72.9 (4.3) | 14.5 (1.3) | 14.4 (1.1) | 45.6 (2.3) | 46.3 (1.7) | 16.2 (3.2) | 16.2 (3.4) |
| Range | 6.7-13.5 | 6.7-13.3 | 66.5-81.5 | 47.2-79.6 | 13-19 | 12-17 | 37.7-52 | 42.5-50 | 9.9-24.1 | 10.3-38.2 |
| Z-score, Mean (SD) | -0.3 (1.4) | -0.2 (1.5) | -0.8 (1.3) | -0.7 (2.2) | 0.1 (1.1) | -0.0 (0.9) | 0.1 (1.7) | 0.7 (1.4) | 0.3 (1.4) | 0.3 (1.8) |

^1^includes one child with microcephaly ^2^N for Z-scores is less than N for mean and range as Z-score conversions for preterm infants was not possible
